# A Question of Privilege

**Published:** 1898-04-09

**Authors:** 


### A QUESTION OF PRIVILEGE.

A question has recently been raised as to the right of an insurance company to require from the medical man who has attended an insured person before his death, all sorts of details as to the diseases for which he has attended the deceased. The answer seems to us to be quite plain. The company has no right to claim that such a certificate form should be filled up unless it is definitely stated in the agreement between the company and the assured that such a certificate shall be given; and even then the medical man is by no means bound to fill it up, although he would be justified in so doing. Where, however, no such agreement has been made, we do not think that a medical man is justified in stating on the certificate facts which have come to his knowledge in his professional capacity. Of course we are not speaking here of the statutory certificate of the cause of death. The law makes ample provision for the protection of the company. If it refuses the claim the matter comes before the courts, and then the doctor can be made to speak. The law is supreme, but there is no obligation to say a word except by direction of the judge.

